# Decodability of Reward Learning Signals Predicts Mood Fluctuations

**DOI:** 10.1016/j.cub.2018.03.038

**Published:** 2018-05-07

**Authors:** Eran Eldar, Charlotte Roth, Peter Dayan, Raymond J. Dolan

**Affiliations:** 1 Max Planck University College London Centre for Computational Psychiatry and Ageing Research, London WC1B 5EH, UK; 2 Wellcome Trust Centre for Neuroimaging, University College London, London WC1N 3BG, UK; 3 Gatsby Computational Neuroscience Unit, University College London, London W1T 4JG, UK

**Keywords:** mood, reward, ecological momentary assessment, wearable sensors, reinforcement learning, prediction errors

## Abstract

Our mood often fluctuates without warning. Recent accounts propose that these fluctuations might be preceded by changes in how we process reward. According to this view, the degree to which reward improves our mood reflects not only characteristics of the reward itself (e.g., its magnitude) but also how receptive to reward we happen to be. Differences in receptivity to reward have been suggested to play an important role in the emergence of mood episodes in psychiatric disorders [1, 2, 3, 4, 5, 6, 7, 8, 9, 10, 11, 12, 13, 14, 15, 16]. However, despite substantial theory, the relationship between reward processing and daily fluctuations of mood has yet to be tested directly. In particular, it is unclear whether the extent to which people respond to reward changes from day to day and whether such changes are followed by corresponding shifts in mood. Here, we use a novel mobile-phone platform with dense data sampling and wearable heart-rate and electroencephalographic sensors to examine mood and reward processing over an extended period of one week. Subjects regularly performed a trial-and-error choice task in which different choices were probabilistically rewarded. Subjects’ choices revealed two complementary learning processes, one fast and one slow. Reward prediction errors [17, 18] indicative of these two processes were decodable from subjects’ physiological responses. Strikingly, more accurate decodability of prediction-error signals reflective of the fast process predicted improvement in subjects’ mood several hours later, whereas more accurate decodability of the slow process’ signals predicted better mood a whole day later. We conclude that real-life mood fluctuations follow changes in responsivity to reward at multiple timescales.

## Highlights

• Choices in a week-long reward learning task reveal slow- and fast-learning processes
• Both processes’ prediction errors can be decoded from EEG and heart-rate responses
• Greater fast-process decodability predicts positive mood change a few hours later
• Greater slow-process decodability predicts positive mood change one day later

## Teaser

In a week-long smartphone experiment, Eldar et al. show that reward-prediction errors indicative of fast and slow reward-learning processes can be decoded from EEG and heart-rate signals. Moreover, fast and slow mood fluctuations are predicted by how well fast and slow learning can be decoded—positive mood changes follow greater decodabilities.

## Results and Discussion

**Figure 1:**
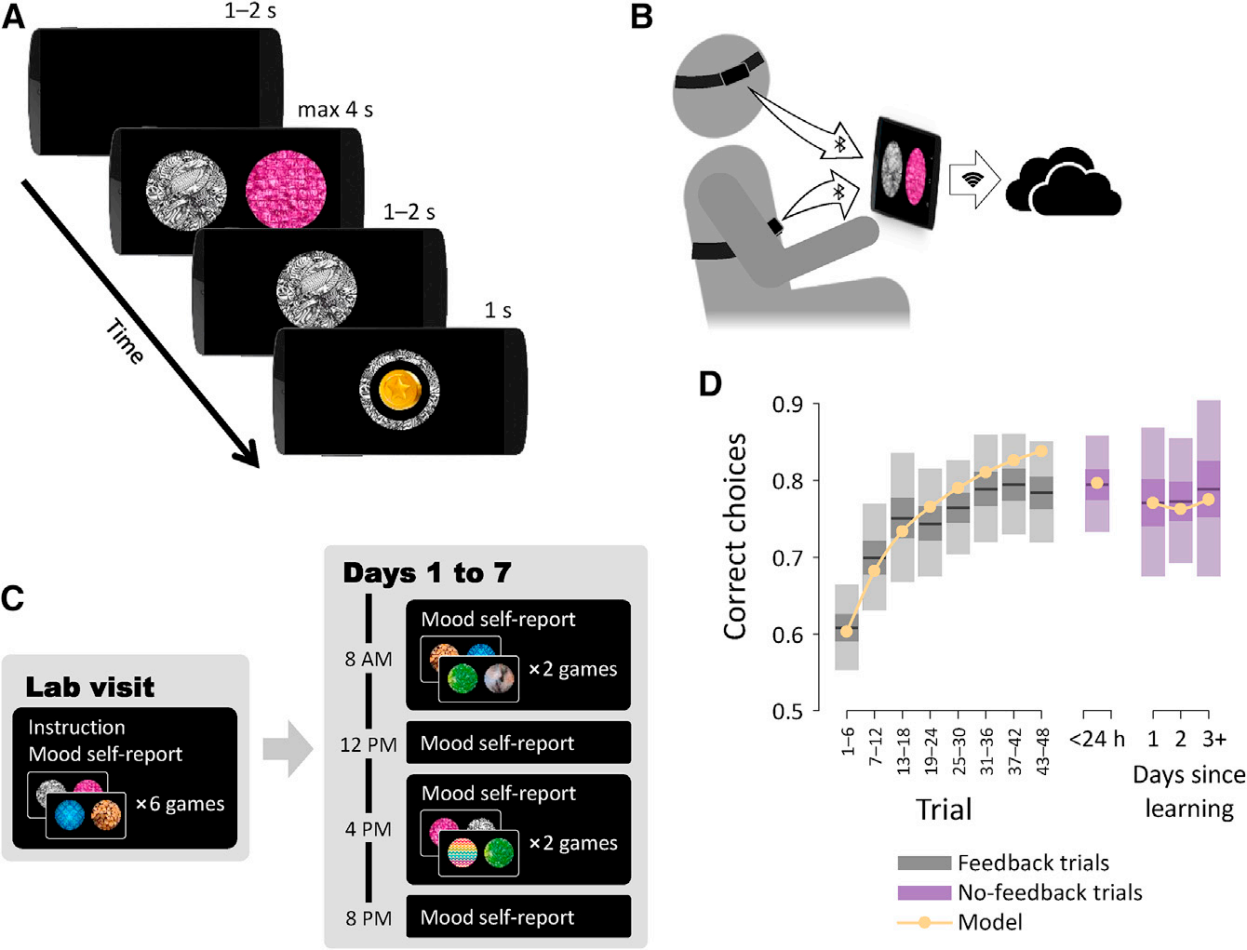
Experimental Task (A) Subjects chose between two images and either received or did not receive a coin reward depending on the probability associated with the chosen image. Each game included 48 such feedback trials, as well as 24 trials in which outcomes were not revealed (no feedback trials). Every image first appeared on two consecutive sessions with feedback and thereafter only appeared again on no feedback trials. (B) Subjects performed the experimental task on a smartphone while a chest strap and a headband transmitted heart rate and EEG signals to the phone. Data were then uploaded to a dedicated online server. (C) Following an initial lab visit, subjects performed two experimental task sessions every day and reported their mood four times a day. (D) Task performance computed as the proportion of choices of the image associated with a higher reward probability. Also shown is simulated performance of the computational model (see Figure 2). Performance on ‘no feedback’ trials is shown as function of the time that passed since images appeared with feedback. Shaded areas: SEM (dark) and SD (light). n=10 subjects.

10 human volunteers reported their mood four times a day, and performed a reward learning task twice a day, for a period of one week (Figures 1A–1C). Overall, each subject completed a total of 2,316 task trials. On each trial, subjects chose between two available images and were rewarded with a coin depending on a reward probability associated with the chosen image. Each of the two daily sessions included two “games” in which trials involving choices between new images and explicit reward feedback (“feedback” trials) were interleaved with trials involving choices between familiar images taken from previous sessions (“no feedback” trials). In each game, the feedback trials involved a set of three images associated with reward with fixed probabilities of 0.25, 0.50, and 0.75. These probabilities were unknown to the subjects and thus could only be learned by trial and error based on obtained rewards. Thus, subjects’ performance improved over the course of each game such that by the end of the game, they were choosing the image associated with a higher reward probability 78% of the time (±2% SEM). We tested how well subjects maintained the information they had learned in previous sessions by means of no-feedback trials. In these trials, rewards were administered as before but were not shown to the subject so as to avoid further reward-based learning. Subjects maintained comparable levels of performance on these no-feedback trials even when outcomes associated with the images had not been observed for a period of 3 days (Figure 1D).

**Figure 2:**
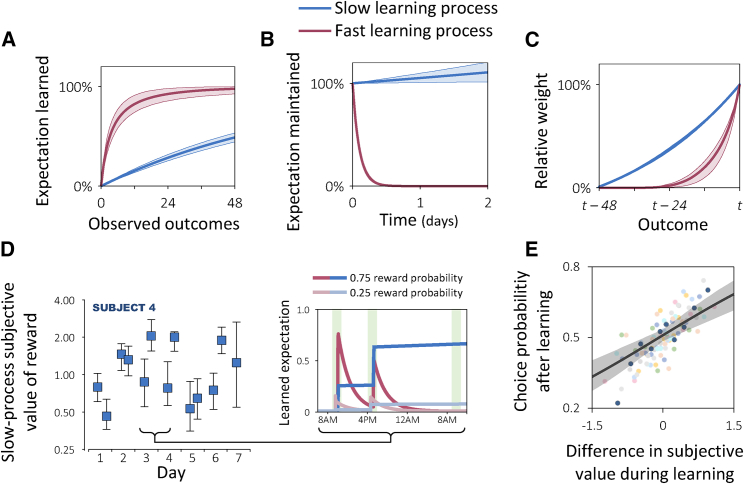
Fast and Slow Learning Subjects’ choices were best explained by a model that involves two simultaneous learning processes. n=10 subjects. see STAR Methods and Figure S1 for details of modeling procedures. (A) Expectations learned by the two processes given a fixed repeating outcome. Shaded areas indicate spread across subjects (95% interval of fitted group-level distribution). (B) Decay of expectations as a function of time. (C) Weights assigned by the two processes to different outcomes as a percentage of the most recent outcome’s weight. (D) Subjective value of reward for the slow process in an exemplar subject. Inset shows the subject’s expectations for two images for the slow and fast processes over three sessions (green shading). The images appeared with feedback only in the first two sessions. Error bars: 95% credible interval. (E) Image choice probability in ‘no feedback’ trials as a function of the subjective value of reward during learning about the image, minus the subjective value during learning about the alternative image. For visualization, trials were divided into ten quantiles of subjective value differences (each circle represents 10% of a subject’s trials). Subjects are color-coded (dark blue: subject from [D]). Choice probabilities are corrected for the number of reward outcomes observed for each image. Shaded areas: 95% bootstrap CI.

Relatively little is known about how learning over a timescale of minutes translates to a timescale of days [19], but previous work suggests that humans might learn separately about short and long timescales [20, 21]. Therefore, we first asked whether the choices a subject made over the course of the experiment reflected a single learning process or, alternatively, an additive combination of multiple learning processes that operate over different timescales. In particular, we compared several learning models in terms of how well each model fitted subjects’ choices (see STAR Methods; Figure S1). We found that subjects’ choices were best explained by a combination of two learning processes: one that learns quickly but forgets what it has learned by the end of the day and another that learns slowly and does not forget (Figures 2A–2C). In fact, the latter, slower process was best fitted with a negative expectation decay parameter, which entails that what is learned is not only maintained but is actually consolidated or amplified [22] at an average rate of 5.4% per day (Figure 2B). Indeed, the impact of the rewards associated with an image on subjects’ choices increased with time even though during this time the image was not associated with additional rewards (${\bar{\beta }}_{\text{rwd }\times \text{ time}}=0.12\pm 0.05$, $p_{\text{boostrap}}=0.04$, logistic regression of choices between images about which subjects learned at least one day ago as a function of sum of observed rewards, the average time since these rewards were observed, and their interaction). Importantly, the multi-timescale dynamics captured by the two-process model could not be captured using more complex models that allow for multiple timescales but learn only a single set of expectations (BIC difference of 1377; Figure S1B). Thus, the modeling results revealed fast- and slow-learning processes, each with its own set of learned expectations.

Building on the insight afforded by our learning model about how subjects solved the task, we next asked how subjects’ processing of rewards changed from session to session. We first tested variants of the model in which different aspects of the fast- or slow-learning processes were allowed to vary from session to session. These aspects included the learning rates, the decision temperatures, and the subjective value of reward outcomes. We found that variability in subjects’ choices across the experiment was best explained by assuming that, for the slow (but not the fast) process, the subjective value of a coin obtained in one session could differ from that of an identical coin obtained in a different session (Figure S1C; Figure 2D). These fluctuations in subjective value during learning explained subjects’ later preferences when they were asked to choose between images from different sessions (Figure 2E).

This session-by-session behavioral measure of reward sensitivity, which is based on subjects’ choices, did not significantly correlate with subsequent mood changes or with current mood ($p_{\text{bootstrap}}>0.1$; see STAR Methods). This is despite the fact that subjects’ reported mood did vary considerably over the course of the week (mean range 61%; Figure S2B). However, receptivity to reward has at least two aspects. First, there is *sensitivity* [1], which is reported above and which maps objective reward values into subjective utilities. Second, there is *responsivity*, which reflects the attention paid to the dimension of reward and which we operationalized as the degree to which physiological responses (e.g., Figure S3) reflect signals indicative of reward processing. Reward prediction errors, in particular, have been suggested to mediate the emotional impact of reward [3, 23, 24, 25], and thus, we next examined whether the reward prediction errors that drove learning according to the model were manifested in subjects’ physiological responses and whether this physiological responsivity provided a measure more closely reflective of the dynamics underlying mood changes.

**Figure 3:**
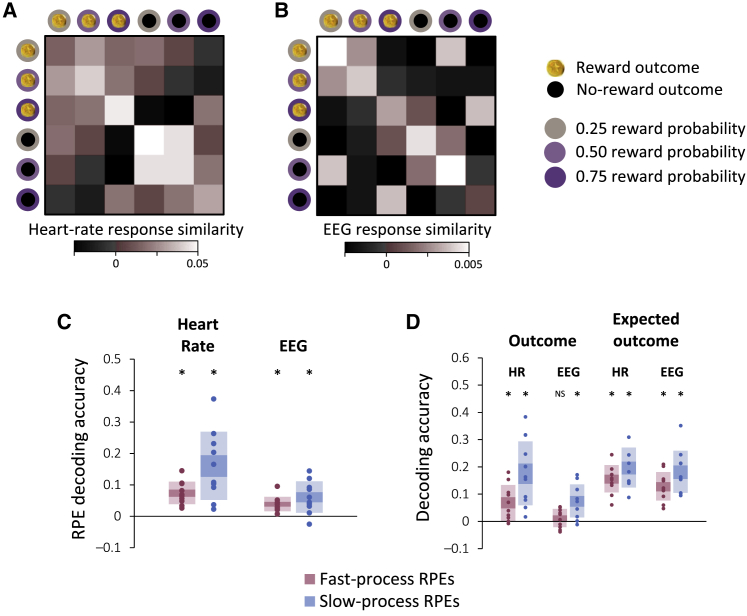
Heart-Rate and EEG Responses to Outcomes n=10 subjects. (A) Similarity between heart-rate responses recorded in different sessions following different types of outcomes. Similarity was computed as the average temporal (Pearson) correlation between heart-rate responses for six types of outcomes: reward and no-reward outcomes following choices of images associated with a 0.25, 0.50, or 0.75 reward probability. Similarity was computed separately for each subject and then averaged across subjects. (B) Similarity between EEG responses recorded in different sessions following different types of outcomes. See Figure S3 for time courses of heart-rate and EEG responses for exemplar subjects. (C) Reward prediction errors (RPEs) of the fast- and slow-learning processes were decoded from the physiological response to outcomes. The y axis denotes decoding accuracy, computed as the correlation between decoded and actual values. RPEs were derived using the model (see Figure 2) and decoded with cross-validated support vector regression (see STAR Methods). (D) Correlation between actual and decoded outcomes and between actual and decoded expectations for the slow and fast processes. In (C) and (D), circles correspond to individual subjects. Shaded areas: SEM (dark) and SD (light). ^∗^: $p_{\text{permutation}}<0.01$, NS: $p_{\text{permutation}}=0.2$.

To examine this possibility, we first tested whether physiological responses were consistently modulated by the two elements that compose a prediction error—namely, actual and expected outcome [17, 18]. For this purpose, we computed the average time series of the heart rate (from 1 s before to 10 s after each outcome) and of the EEG signal (from 0.5 s before to 1.5 s after each outcome) during each session for each of six types of outcomes: reward and no-reward outcomes where reward probability was 0.25, 0.50, or 0.75. We then measured the similarity between responses from different sessions (see STAR Methods), and, indeed, we found that physiological responses to the same type of outcome were more similar than responses to different types of outcome (Figures 3A and 3B; Heart rate: ${\bar{r}}_{\text{same}}\;=\;0.038$, ${\bar{r}}_{\text{different}}\;=\;-0.001$, $p_{\text{bootstrap}}\;=\;10^{-6}$; EEG: ${\bar{r}}_{\text{same}}\;=\;0.005$, ${\bar{r}}_{\text{different}}\;=\;-0.001$, $p_{\text{bootstrap}}\;=\;10^{-7}$).

This result indicates that the components of the reward prediction error were consistently reflected in subjects’ physiological responses. However, this analysis can be improved on in several ways through the medium of the model. First, subjects’ expectations of each image were not fixed. Instead, they were dynamically updated as a function of observed outcomes, and the model provides trial-by-trial estimates of these changing expectations. Second, the model indicated that subjects maintained two sets of expectations, and therefore, two sets of prediction errors should be reflected in physiological responses. Third, the model indicated that for the slow-learning process, the subjective value of reward also varied, implying that prediction errors should be computed with respect to this subjective value.

To account for these nuances in subjects’ learning, we derived trial-by-trial prediction errors for each subject from the fast- and slow-learning processes of the model, with parameters fitted to that subject’s choices. We then measured the degree to which each series of prediction errors was reflected in subjects’ physiological responses by attempting to decode them from the physiological data using support vector regression with radial basis functions. The degree of success in decoding using this nonlinear method provided us with a single measure of physiological reward prediction error signaling that accounts not only for simple effects of intensity, but also for individualized changes in the shape, timing, and sign of the physiological response. To prevent overfitting in this procedure, we decoded prediction errors for each trial using a decoder trained on a separate set of trials (i.e., using nested cross-validation), and we compared the resultant decoding accuracy to that obtained by applying the same procedure to randomly permuted data (see STAR Methods).

We found that both heart-rate and EEG responses to outcomes reflected the predictions errors generated by the slow- and fast-learning processes of the model (Figure 3C). Moreover, we found that the two components that compose prediction errors, namely actual and expected outcomes, were each separately decodable from subjects’ physiological responses (Figure 3D). In addition, combining the decoding from the heart-rate and the EEG responses yielded statistically significant decoding accuracy ($p_{\text{premutation}}<0.05$) for each individual subject for the slow process, and for 8 out 10 subjects for the fast process. Since both processes learned from the same series of choices and outcomes, and thus their prediction errors were correlated ($\bar{r}=0.75$, $p_{\text{bootstrap}}<10^{-5}$), we tested whether decoded prediction errors specifically reflected the learning process from which they were derived. For this purpose, we examined the correlation between the decoded prediction errors of one process and the prediction errors of the other process. We found no such correlations for either the heart-rate or EEG responses (all $\bar{r}<0.006$, $p_{\text{bootstrap}}>0.6$). Interestingly, by computing decoding accuracy separately for each experimental session, we found that decoding from heart rate was not significantly correlated across sessions with decoding from EEG, for either the fast ($\bar{r}=0.09$, $p_{\text{bootstrap}}=0.34$) or slow ($\bar{r}=0.03$, $p_{\text{bootstrap}}=0.71$) processes. However, for each of the two physiological sources, decoding accuracies for the fast and slow processes were correlated with one another ($\text{Heart rate}:\;\bar{r}=0.28$, $p_{\text{bootstrap}}<10^{-6}$; $\text{EEG}:\;\bar{r}=0.24$, $p_{\text{bootstrap}}=10^{-5}$).

**Figure 4:**
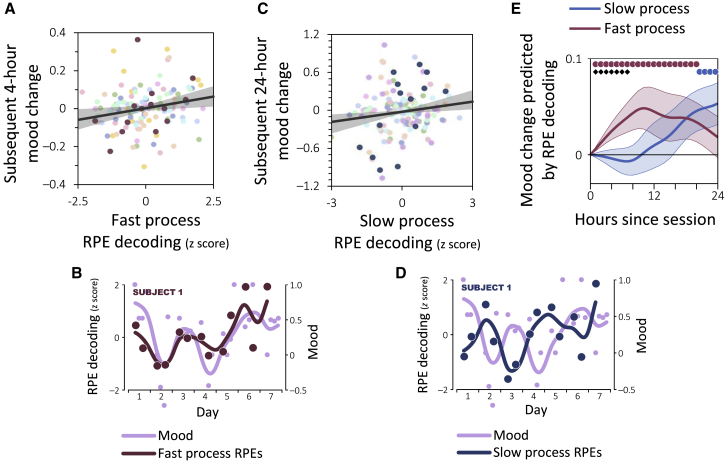
RPEs Evident in EEG and Subsequent Mood Changes n=10 subjects. (A and C) Change in self-reported mood as a function of RPE decoding accuracy for the fast- (A) and slow- (C) learning processes. Decoding accuracy was computed separately for each session (denoted by circles). Subjects are color coded, with the subject from (B) and (D) highlighted in dark red (A) and dark blue (C). (B and D) Relationship between RPE decoding and mood in an exemplar subject for the fast- (B) and slow- (D) learning processes. Shifts in mood follow the fast process’s PE signaling almost immediately but substantially lag the slow process’s signals. (E) Average change in mood following each experimental session as a function of reward PE decoding. Magnitude of change is shown per one standard deviation of decoding accuracy. •: difference from zero, ◆: difference between processes (*p*_corrected_ < 0.05). Shaded areas: SEM. See Figures S2C and S2D for a similar analysis with respect to heart-rate responses and reward sensitivity.

We next tested whether more robust physiological reward-prediction-error signaling (i.e., high responsivity to reward) was followed by improvement in subjects’ mood. For this purpose, we tested the relationship between the decodability of prediction errors in a given experimental session and how subjects’ self-reported mood changed following the session. Thus, we examined changes in self-reported mood 4 hr following each session, when subjects were next asked to report their mood. In addition, to control for possible diurnal variations in mood [23], we also examined mood 24 hr following each session. Since we were agnostic as to which physiological source (heart rate or EEG) would best reflect future mood change and what timescale of mood change would be reflected (4 or 24 hr), we corrected for the four possible combinations using Bonferroni correction for multiple comparisons. We found that EEG signals reflecting the reward prediction errors derived from the fast process predicted 4-hr mood changes ($\bar{\beta }=0.025\pm 0.009$, $p_{\text{bootstrap}}=0.003$, linear regression controlling for current mood; Figures 4A and 4B), whereas EEG signals derived from the slow process predicted 24-hr mood changes ($\bar{\beta }=0.053\pm 0.021$, $p_{\text{bootstrap}}=10^{-4}$; Figures 4C and 4D). In both cases, higher prediction-error decodability predicted more positive mood, and lower decodability predicted worse mood. Neither of these predictive relationships reflected fluctuations in task performance ($p_{\text{bootstrap}}<0.009$ when including task performance as a control regressor). In contrast, the fast-process signals did not predict 24-hr mood changes ($\bar{\beta }=0.018\pm 0.022$, $p_{\text{bootstrap}}=0.4$; difference from slow process: $p_{\text{bootstrap}}=0.001$), nor did the slow-process signals predict 4-hr mood changes ($\bar{\beta }=-0.004\pm 0.009$, $p_{\text{bootstrap}}=0.7$; difference from fast process: $p_{\text{bootstrap}}=0.18$). Thus, we found a significant interaction between the timescale of the learning process and the timescale of subsequent mood changes ($p_{\text{bootstrap}}\;=\;0.001$). A complementary analysis involving all time lags up to 24 hr showed similar results (Figure 4E). No such relationship was found between mood changes and the heart-rate signals ($p_{\text{bootstrap}}>0.1$), which were also not correlated with the EEG signals ($p_{\text{bootstrap}}>0.1$). These findings establish a striking double dissociation between fast- and slow-learning EEG signals in predicting fast and slow mood fluctuations.

We have shown that responsivity to reward, manifesting as reward prediction error signals in EEG, is predictive of subsequent mood changes. Moreover, this predictive relationship reflects multiple timescales in both reward learning and the dynamics of mood. The finding of multiple timescales adds to previous theoretical accounts of mood as reflecting changes in the availability of reward [3, 26], suggesting that fast and slow changes in mood track short-term and long-term changes in this availability. Future research could investigate whether the distinction between fast- and slow-learning processes evident here reflects the operation of separate brain systems [27, 28] or complex multi-timescale dynamics arising within the same neural population [21, 29]. More importantly, our results show that people’s responsivity to reward prediction errors changes from day to day and that greater responsivity is followed by elevated mood, whereas lower responsivity is followed by depressed mood. These findings suggest that day-to-day changes in reward responsivity may play an important role in the generation of natural daily mood fluctuations.

We found leading indicators of changes in mood over two timescales. The precise psychological nature of these indicators, which are based on EEG decoding accuracy, remains to be determined. In the present experiment, these indicators did not consistently reflect task performance (correlation with accuracy: $p_{\text{bootstrap}}\geq 0.27$) nor long-term learning (correlation with model parameter ψ: $p_{\text{bootstrap}}\geq 0.34$). Thus, the processes that impair the accuracy of decoding, the influence of those processes on momentary computations involving reward, and the interaction between these processes and the internal thoughts and external events that can influence subsequent mood become tempting targets for future investigation. Importantly, we note that our results do not rule out the possibility that similar mood-predicting signals also manifest in heart-rate responses or choice behavior (Figures S2C and S2D).

Our EEG measures provide an ecological and scalable means to assess fluctuations in reward responsivity that might prove useful for investigating and predicting how pathological mood episodes evolve—for instance, in major depression and bipolar disorder. The therapeutic effect of existing drug and talk therapies is suggested to reflect their impact on patients’ processing of reward [30, 31], and this may serve as a target for the development of new therapeutic approaches.

## STAR★Methods

### Key Resources Table

**Table:** 

REAGENT or RESOURCE	SOURCE	IDENTIFIER
**Software and Algorithms**		

MATLAB 2016a	MathWorks	RRID: SCR_001622
LIBSVM	[32]	RRID: SCR_010243
FieldTrip	[33]	RRID: SCR_004849

### Contact for Reagent and Resource Sharing

Further information and requests for resources or raw data should be directed to and will be fulfilled by the Lead Contact, Eran Eldar (e.eldar@ucl.ac.uk).

### Experimental Model and Subject Details

#### Subjects

10 human subjects, aged 20 to 29, 8 female, completed the experiment. Subjects were recruited from a subject pool at University College London (UCL). Before being accepted to the study, each subject was asked whether they satisfy any of the study’s inclusion or exclusion criteria. Inclusion criteria included fluent English and possession of an Android smartphone that could connect to wearable sensors via Bluetooth Low Energy. Exclusion criteria included age (younger than 18 or older than 30), impaired color discrimination, use of psychoactive substances (e.g., psychiatric medications), and current neurological or psychiatric illness. Subjects received £10 per day for participation and 6 pence for each coin they collected in the experimental task, which together added up to an average sum of £151.04 (±£1.77 SD). The experimental protocol was approved by the University of College London local research ethics committee, and informed consent was obtained from all subjects.

### Method Details

#### Experimental design

To study the temporal relationship between reward responsivity and mood, we had subjects regularly report their mood, while also performing a reward learning experimental task, over a period of one week, using a mobile phone platform that we developed for this purpose. Since we aimed to characterize a longitudinal process that manifests in most people, we opted to study a relatively small group of 10 subjects but to collect a very large dataset from each. Thus, each subject made 2316 choices in the experimental task while their physiological responses were being recorded. Due to the novelty of the experimental measures, this sample size was not determined based on a quantitative power analysis. However, the amount of data collected from each subject was an order of magnitude greater than the amount of data that comprise a typical learning study. Due to the size of this dataset, we exercised particular caution in determining whether subjects’ physiological responses reflected reward prediction errors. To this end, we separated between training and testing data, we tested statistical significance using permutation tests, and we replicated the finding of physiological prediction error signals separately for each individual subject (see Physiological responses decoding below). In addition, due to the relatively small number of subjects, we only tested main effects across the whole study sample.

#### Mobile platform

To allow a longitudinal study of reward learning processes, associated physiological responses, and their interaction with mood fluctuations, we developed an app for Android smartphones using the Android Studio programming environment (Google, Mountain View, CA). The app asks users to perform experimental tasks according to a pre-determined schedule, while it records electroencephalographic (EEG) and heart rate signals derived from wearable sensors connected using Bluetooth. Additionally, we equipped the app with additional features designed to probe changes in a person’s mental state, including regular mood self-report questionnaires and life events and activities logging. Motor activity was tracked via the phone’s accelerometer and global positioning system (GPS). Subjects also completed a temperamental trait questionnaire. All behavioral and physiological data were saved locally on the phone as SQLite databases (The SQLite Consortium), which were regularly uploaded via the phone’s data connection to a dedicated cloud storage space.

#### Daily schedule

Subjects first visited the lab to receive instructions, test the app on their phones, and try out the experimental task (see Initial lab visit section below). Starting from the next day, subjects performed two experimental sessions a day, one in the morning and one in the evening, over a period of 7 days. Each session began with a 5-minute heart rate measurement during which subjects were asked to remain seated, report their mood, and perform a circle drawing task (see details below). Following this, subjects put on the EEG sensor and played two games of the experimental task. The app allowed subjects to perform the morning session from 8AM and the evening session from 4PM. In addition, subjects were asked to report their mood twice more, at 12PM and 8PM. Subjects were allowed to adjust the timing of the sessions according to their daily schedule, but were required to ensure a gap of at least 4 hr between successive sessions. On average, subjects performed the morning session at 9:06AM (mean SD ± 25 min) and the evening session at 5:21PM (mean SD ± 32 min), and provided additional mood self-reports at 12:44PM (mean SD ± 19 min) and 20:23PM (mean SD ± 60 min). One subject was not able to perform the experiment on Day 2, and thus all her subsequent tasks were postponed by one day.

#### Experimental task

To test for fluctuations in reward processing, we had subjects perform regularly a trial-and-error learning task over a period of one week. On each trial, subjects chose from one of two available images, and then collected a coin reward with a probability associated with the chosen image (Figure 1A). Each game consisted of 48 such trials involving a set of 3 images with reward probabilities of 0.25, 0.5 and 0.75. The probabilities were never revealed to the subjects, though subjects were instructed that each image was associated with a fixed probability of reward. Subjects played four games a day, two during the morning session and two during the evening session.

To examine changes in subjects’ learning throughout the week, we had each image appear with reward feedback only in two successive sessions. This way, subjects learned about each given image during a specific part of the week, and this allowed us to probe fluctuations in the effect of learning by later asking subjects to choose between images they had learned about during different parts of the week. To prevent new learning during this probing, outcomes were not revealed on such trials but subjects were informed that they would be rewarded for their choices as before (at the end of the entire experiment). Each game included 24 such no-feedback trials (every 3^rd^ trial), 12 of which involved choosing between images associated with the same reward probability. The no-feedback trials primarily allowed us to measure subjects’ rate of forgetting, since they involved familiar images re-appearing following variable lags after subjects had learned about them. In addition, the interleaving of feedback trials involving new images with no-feedback trials involving familiar images allowed us to dissociate fluctuations in how subjects learned from fluctuations in how subjects formed their decisions (see Modeling sections below).

In the first two days, familiar images were taken from the session performed during the initial lab visit. Thereafter, familiar images were those subjects learned about during the week. The app dynamically populated the no-feedback trials of each game to ensure the following criteria: 1. No pair of images appeared more than once in a given game. 2. The app prioritized pairs of images that had previously appeared fewer times. 3. Out of pairs that had appeared a similar number of times, the app prioritized pairs of images about which the subject learned in dissimilar moods. To compute mood during learning about a given image, the app computed the average timing of all revealed outcomes associated with the image and linearly interpolated between the mood self-reports preceding and following this timing. The last 4 games of the experiment consisted solely of no-feedback trials involving familiar images, with 48 such trials per block. Thus, the evening session on Day 7 consisted of 3 such games, and another such game was played prior to that in Day 7’s morning session.

#### Modeling: learning and forgetting

To identify the computations that guided subjects’ choices in the experimental task we compared a set of computational models in terms of how well each model fitted subjects’ choices. We were first interested in determining how subjects learned from the outcomes associated with each image, and whether the learned information decayed as a function of time.

To this end, we compared the following four models:

Model 1 (fixed learning; Equations 1, 2, and 3) learns the expected value of each image by adjusting its expectation following each outcome as follows:

(Equation 1) $$Q_{t+1}\left(s_{t,c_{t}}\right)=Q_{t}\left(s_{t,c_{t}}\right)+\eta \delta_{t},$$

where $s_{t,c_{t}}$ is the image chosen at trial t, $Q_{t}\left(s\right)$ is the expected outcome for image s (initialized as $Q_{0}\left(s\right)=0$), η is a fixed learning rate parameter between 0 and 1, and $\delta_{t}$ is the prediction error at trial t:

(Equation 2) $$\delta_{t}=R_{t}-Q_{t}\left(s_{t,c_{t}}\right),$$

computed as the difference between the outcome and the expected outcome (a reward outcome corresponds to R=1 and no-reward to R=0). On each trial, the model chooses either the left image $\left(c_{t}=1\right)$ or the right image $\left(c_{t}=2\right)$, according to the expectations it has learned:

(Equation 3) $$\text{p}\left(c_{t}=i\right)=\frac{e^{\beta Q_{t}\left(s_{t,i}\right)}}{\sum_{j=1}^{2}e^{\beta Q_{t}\left(s_{t,j}\right)}},$$

where $s_{t,1}$ and $s_{t,2}$ are the left and right images, respectively, the subject can choose on trial t, and β is an inverse temperature parameter.

Model 1 has a fixed learning rate, and thus, it assigns greater weight to more recent outcomes (i.e., ‘leaky integration’). In contrast, Model 2′s (dynamic learning; Equations 2, 3, 4, and 5) learning rate changes as a function of the number of observed outcomes $\left(N_{t}\right)$ for the chosen image:

(Equation 4) $$Q_{t+1}\left(s_{t,c_{t}}\right)=Q_{t}\left(s_{t,c_{t}}\right)+\alpha_{t}\delta_{t},$$

(Equation 5) $$\alpha_{t}=\frac{1}{\varepsilon +N_{t}\left(s_{t,c_{t}}\right)},$$

where ε is a free parameter that determines the initial learning rate. Here, the learning rate gradually decreases asymptotically toward zero so as to compute an average of observed outcomes in which all outcomes are similarly weighted. ε>0 slows down initial learning, and its impact is similar to that of a prior expectation that all expected outcomes equal $Q_{0}$, with the precise value of ε reflecting the strength of this prior.

Model 3 (‘fixed + dynamic learning’; Equations 2, 3, 4, and 6) combines Models 1 and 2 in that its learning rate is composed of fixed and changing components, implying that the learning rate gradually decreases to an asymptote that is larger than zero:

(Equation 6) $$\alpha_{t}=\eta +\frac{1-\eta }{\varepsilon +N_{t}\left(s_{t,c_{t}}\right)},$$

Model 4 (‘fixed learning + decay’; Equations 1, 2, 3, and 7), Model 5 (‘fixed & dynamic learning + decay’; Equations 2, 3, 4, 5, and 7), and Model 6 (fixed & dynamic learning + decay’; Equations 2, 3, 4, 6, and 7) are similar to Models 1, 2, and 3, except that expectations decay back to zero as a function of time, both during and in between sessions. To implement this decay, we updated all model expectations at the beginning of every trial as follows:

(Equation 7) $$Q_{t}\left(s\right)\leftarrow Q_{t}\left(s\right)e^{-\gamma \left(T_{t}-T_{t-1}\right)\;},$$

where $T_{t}$ is the time at trial t, measured in units of days, and γ determines the rate of decay.

Out of these six models, we found that the model that best fitted subjects’ choices was Model 6 (‘fixed & dynamic learning + decay’), and thus, in the next step we tested variants of this basic model.

#### Modeling: multiple timescales

Since learning within a single session, over a timescale of minutes, might involve different processes than learning over a whole week, we tested whether subjects’ choices could be better explained by allowing the model to operate over two different timescales. For this purpose, we compared Model 6 with a combination of two such models, each with its own set of expectations (Q and $Q^{'}$) and parameters (η and $\eta^{'}$, ε and ε, γ and $\gamma^{'}$,β and $\beta^{'}$). This combined model (Model 7, ‘Two dynamic-learning processes’; Equations 2, 4, 6, 7, and 8) simultaneously learns two sets of expectations, updating both in the same manner but with different learning and decay rates. Importantly, in the iterative model fitting procedure described below (Model Fitting subsection), the learning and decay rate parameters of the two processes spontaneously differentiated so as to form one fast process and one slow process. The model forms its decisions by combining the two sets of expectations:

(Equation 8) $$\text{p}\left(c_{t}=i\right)=\frac{e^{\beta Q_{t}\left(s_{t,i}\right)+\beta^{\text{'}}Q_{t}^{\text{'}}\left(s_{t,i}\right)}}{\sum_{j=1}^{2}e^{\beta Q_{t}\left(s_{t,j}\right)+\beta^{\text{'}}Q_{t}^{\text{'}}\left(s_{t,j}\right)}}.$$

Model 8 (’two processes: dynamic + fixed; Equations 1, 2, 4, 6, 7, and 8) is a variant of Model 7, also involving two independent learning processes, except that in this model one of the processes has a fixed learning rate (as in Equation 1).

Since Models 7 and 8 fitted subjects’ choices significantly better than a single-process model, we next tested whether an additional set of expectations was indeed necessary. To this end, we tested whether subjects’ choices can be better fitted with more complex single-process algorithms that allow for multiple timescales but only maintain a single set of expectations. Specifically, we designed the following four models:

Model 9 (‘single process: multiple learning dynamics’; Equations 2, 3, 4, 7, and 9) allows for more complex learning-rate dynamics, since its learning is composed of one fixed component (η) and two separate dynamic components (ε and $\varepsilon^{'}$):

(Equation 9) $$\alpha_{t}^{\text{'}}=\eta +\frac{\omega \left(1-\eta \right)}{\varepsilon +N_{t}\left(s_{t,c_{t}}\right)}+\frac{\left(1-\omega \right)\left(1-\eta \right)}{\varepsilon^{\text{'}}+N_{t}\left(s_{t,c_{t}}\right)},$$

where 0<ω<1.

In Model 10 (‘single process: multiple forgetting dynamics’; Equations 2, 3, 4, 6, and 7) expectations decay at a different rate within (γ) and between ($\gamma^{'}$) sessions and, in addition, the expected value of each image is multiplied by a positive factor ($\gamma^{''}$) once learning about the image concludes.

Model 11 (‘single process: multiple decision temperatures’; Equations 2, 3, 4, 6, and 7) forms its decisions with different inverse temperature parameters (β and $\beta^{'}$) depending on whether the trial involves new images (β; ‘feedback’ trials involving images the model is still learning about) or familiar images ($\beta^{'}$; ‘no feedback’ trials involving images about which learning has concluded).

Model 12 (‘single process: two full sets of parameters’; Equations 2, 3, 4, 7, and 9) combines all of the enhancements featured by Models 12 to 14.

We found that none of the single-process models fitted subjects’ choices nearly as well as the best two-process model (Model 8) and therefore we used Model 8 as a basis for the last model comparison.

#### Modeling: session-to-session variability

In the models described so far, all parameters of an individual subject are sampled from a group-level distribution and remain fixed throughout the subject’s sessions. To test whether (and in what way) a subject performed the task differently in different sessions, we compared Model 8 with six variants of this model in which either the learning rate, or the subjective value of reward outcomes during learning (modeled as $R_{t}^{'}=\psi R_{t}$), or the inverse decision temperature (β), was allowed to vary across sessions for one of the learning processes. For this purpose, for the value of the variable parameter was determined as before, but was then multiplied by a session-specific scaling parameter. The natural logarithm of this scaling parameter was sampled from a subject-specific normal distribution with a zero mean and a standard deviation that was sampled from a group-level gamma distribution.

We found that the model that best fitted subjects’ choices was the model with variable subjective value of reward for the slow process (Model 18). Since this subjective value is learned, it has a lasting impact in future sessions when the probe images are presented without feedback. We used this model for all results displayed in the main text, and we produce its graphical model in Figure S4.

#### Additional alternative models

Along with the model comparisons described above, we tested Model 18 against several additional alternative models that did not fit subjects’ choices as well. These included variants of Model 18 with the addition of a fixed choice bias (iBIC = 21085) or a perseveration bias (iBIC = 21084) [34], or where the expectations of the slow and fast processes are combined to form a single prediction (and thus lead to a single prediction error; iBIC = 21155) [35], a model that makes choices based on sampling of previously observed outcomes [36], where the probability of sampling an observation decays with time according to a power law (iBIC = 24310), a model that allows for asymmetry in the rate of learning from positive and negative prediction errors (iBIC = 21083) [37], and a model that uses Bayesian inference to determine which one of the three possible reward probabilities is associated with each stimulus (iBIC = 24022). Equations describing these algorithmic elements are provided elsewhere.

#### Heart rate data collection

Inter-heart-beat intervals were recorded using a Polar H7 chest strap (Polar Electro, Kempele, Finland). The chest strap senses and analyzes electrocardiographic (ECG) signals, and reports the detected inter-beat (R-R) intervals as well as a derived heart rate measurement once every second via Bluetooth Low Energy (BLE). Its measurements have been shown to be highly reliable in comparison with clinical ECG (error rate lower than 0.01%; intra-class correlation coefficient (ICC) > 0.97) [38]. To ensure that subjects started the experimental task at a relatively similar state of rest, subjects wore the heart rate sensor 5 min prior to each session during which a resting heart rate measurement was taken. Subjects were asked to remain seated throughout this time as well as while performing the task. The app allowed subjects to perform the experimental task only while heart-beat intervals were being received and the sensor’s heart rate measurement was not lower than 30 or higher than 250. Due to communication errors and conflicts between the experimental app and the other apps installed on the subjects’ phones, heart rate data from 5.0% of trials were not saved.

#### Heart rate preprocessing

All data analysis was carried out in MATLAB (Mathworks, Natick, MA). Since the heart rate sensor sends a message once every second, we first identified and corrected the timing of messages that were received with a delay of more than 100 ms. Correction was applied only when the delay affected a single isolated message and thus there was no ambiguity with respect to its correct timing. The timing of each message indicates a window of one second within which the inter-beat intervals reported in the message have concluded. To time heart beats more precisely, we found the timings that best minimize the discrepancy between the cumulative sum of consecutive inter-beat intervals and the timings of the messages containing these intervals. This procedure narrowed down the timing of each heart beat to a 4.5 ms window on average, the center of which was considered as the heart beat’s precise time. We then converted the sequences of precisely timed intervals into unsmoothed 20 Hz heart-rate signals, where the heart rate at any given moment is estimated as the inverse of the corresponding interval. The heart rate response to an outcome in the experimental task was assessed based on the heart rate signal recorded from 1 s preceding the outcome to 10 s following the outcome. This provided one 221-feature vector per outcome. Features were z-scored across trials and used for the decoding analyses (see Decoding below). Heart rate responses for which the standard deviation of the signal across time was higher than 5 times the median standard deviation (0.6% of responses) were considered noisy and excluded from further analysis.

#### EEG data collection

EEG was recorded during the experimental task using Brainlink Lite (Neurosky, Hong Kong), a single-channel 512Hz EEG headband. The headband senses electrical signals via 3 dry electrodes placed on the forehead, and reports 512 raw measurements per second as well as several derived measures via Bluetooth. Signals recorded using similar sensors from the same manufacturer have been shown to successfully discriminate subjects’ cognitive and affective states in a range of scenarios [39, 40, 41, 42, 43]. The app allowed subjects to perform the experimental task only while EEG data were being received and the sensor’s signal-quality assessment was lower than 50 (on a scale of 0 and 100, where lower is better). Due to communication issues and software conflicts, EEG data from 1.0% of the trials were not saved.

#### EEG preprocessing

The EEG response to an outcome in the experimental task was assessed based on the EEG signal recorded from 500 ms preceding the outcome to 1500 ms following the outcome. EEG responses for which the standard deviation of the signal across time was higher than 5 times the median standard deviation (0.3% of responses) were considered noisy and excluded from further analysis. Time-frequency analysis of the EEG responses was performed using the FieldTrip toolbox [33] multitaper method with 4-cycle-long Hanning windows for the following eight frequencies: 1, 5, 9, 13, 17, 21, 25, and 29 Hz. Frequencies higher than 30 Hz were excluded so as to mitigate the effects of muscle artifacts. The resulting time-series were downsampled to 15 Hz, providing one 353-feature vector per outcome. These vectors were *z*-scored across trials and used for the decoding analyses.

#### Physiological responses similarity

We tested how consistently outcomes and expectations affected subjects’ heart rate and EEG responses by examining the degree to which physiological responses from different sessions were correlated. To isolate the effect of outcomes and expectations on physiological responses, we *z*-scored responses within each session across trials, and then computed the average response for 6 types of outcomes: reward and no-reward outcomes following choices of three types of image (reward probabilities 0.25, 0.50 and 0.75). Consistency of both heart rate and EEG responses were measured within and between subjects as the average pairwise temporal correlation between responses to similar types of outcomes from different sessions.

#### Physiological responses decoding

To test whether heart rate and EEG responses to outcomes reflected a subject’s prediction errors (which were inferred using the model from the subject’s choices), we trained and tested support vector machines that decoded these prediction errors from the physiological responses. To avoid over-fitting, training and testing were performed on separate sets of trials following a 5-fold cross validation scheme. Training and testing sets were stratified such that the different sets included similar distributions of prediction errors. This analysis was performed using LIBSVM’s implementation [32] of the ν-SVR algorithm [44], whose parameters were fitted to each training set using a nested 5-fold-cross-validated grid search among the following settings: ν = [0.1 0.2 0.3 0.4 0.5 0.6 0.7 0.8 0.9] and *C* = [0.25 0.5 1 2 4]. Decoding accuracy was computed as the correlation between actual and decoded prediction errors.

#### Mood self-reports

The app regularly asked subjects to rate on an analog scale how well they were feeling (Figure S2A). Naturally, subjects did not report their mood at precisely the same times. Consequently, to assess a subject’s mood at a particular time of interest, we computed a weighted average of all the subject’s mood ratings with weights determined by a Gaussian filter centered on the time of interest with a 4-hour standard deviation (approximating the time between mood reports). In addition, following each mood rating, subjects had to report at least one event or activity that may have affected their mood since the last time they were asked, as well as how strong this effect was and whether it was good or bad. No subject reported that performing the experimental task affected their mood. Finally, subjects were also asked to predict how well they expect to feel over the next several hours.

#### Movement tracking

The app tracked subjects’ movement throughout the week by means of the phone’s accelerometer. Movement data were recorded in terms of number of steps and distance with a temporal resolution of 0.2 Hz (except for one subject whose phone did not allow that). Subjects were asked to carry their phones with them at all times unless they were engaged in an activity that precludes that (e.g., swimming). Movement exceeding 20 m or 20 steps was detected during only 6 games out of the 350 games that subjects played in total.

#### Circle drawing

At the beginning of each session, we asked subjects to trace a circle (15 mm diameter) with their thumb as many times as possible for a period of 30 s. This task was modeled after Mergl et al. [45] who showed that patients with depression differ from healthy controls in the kinematics of their strokes. This raises the possibility that stroke regularity could serve as an implicit measure of a person’s mood state. However, we did not analyze performance on this task since subjects reported that the kinematics of their strokes were significantly affected by how moisturized their hands happened to be at the time (this was not an issue in the original study since there a pen was used for this task).

#### Initial lab visit

Subjects first arrived at the Welcome Trust Centre for Neuroimaging in University College London to receive instructions and have the app installed and tested on their phones. In the lab, subjects played 6 games, each consisting of 48-feedback trials involving a unique 3-image set. In addition, the last three games included 12 no-feedback trials (every 5^th^ trial) involving familiar images from the first three games. Subjects also performed the circle drawing task once in the lab, and filled out one mood self-report and a standardized questionnaire (short version of TEMPS-A) designed to measure five temperamental traits (cyclothymic, dysthymic, irritable, hyperthymic, and anxious) [46]. Before allowing subjects to perform the experiment for a whole week, we verified that subjects succeeded in choosing images associated with higher reward probabilities at above-chance levels, and that the heart-rate and EEG data were recorded and saved to the cloud without significant losses.

### Quantification and Statistical Analysis

#### Model fitting

To fit the parameters of the different models to subjects’ decisions, we used an iterative hierarchical expectation-maximization procedure [47]. We first sampled 10^4^ random settings of the parameters from predefined group-level prior distributions. Then, we computed the likelihood of observing subjects’ choices given each setting, and used the computed likelihoods as importance weights to re-fit the parameters of the group-level prior distributions. These steps were repeated iteratively until model evidence ceased to increase (see Model Comparison below for how model evidence was estimated). This procedure was then repeated with 10^4½^ samples per iteration, and finally with 10^5^ samples per iteration. To derive the best-fitting parameters for each individual subject, we computed a weighted mean of the final batch of parameter settings, in which each setting was weighted by the likelihood it assigned to the individual subject’s decisions. Fractional parameters (η, $\eta^{'}$, ω) were modeled with Beta distributions (initialized with shape parameters a = 1 and b = 1). Expectation decay rates (γ, $\gamma^{'}$) and decision parameters (β, $\beta^{'}$, λ) were initially modeled with normal distributions (initialized with μ = 0 and σ = 1) to allow for both positive and negative effects, but were then re-fitted with Gamma distributions if all fitted values were positive. All other parameters were modeled with Gamma distributions (initialized with k = 1, θ = 1).

#### Session-by-session parameter fits

To estimate the best-fitting setting for ψ for each session of a given subject, we sampled 10^5^ random settings from its posterior distribution given the fitted group-level prior and all of the subject’s choices. We then computed a weighted mean of the 10^5^ parameter settings, where the weight of each setting was determined by the likelihood it assigned to the subject’s choices on all ‘feedback’ trials within the session as well as on ‘no feedback’ trials from subsequent session that involved images about which subjects learned during the session.

#### Trial-by-trial prediction errors

We derived reward prediction errors for each observed outcome by instantiating the model with the parameter settings that best fitted the individual subject’s choices.

#### Model comparison

We compared between pairs of models in terms of how well each model accounted for subjects’ choices by means of the integrated Bayesian Information Criterion (iBIC) [48, 49]. To do this, we estimated the evidence in favor of each model (L) as the mean likelihood of the model given 10^5^ random parameter settings drawn from the fitted group-level priors. We then computed the iBIC by penalizing the model evidence to account for model complexity as follows: iBIC=−2lnL+klnn, where k is the number of fitted parameters and n is the number of subject choices used to compute the likelihood. Lower iBIC values indicate a more parsimonious model fit, and the log Bayes Factor [50] comparing two models can be estimated as their iBIC difference divided in half. We validated this model comparison procedure by generating simulated data using each model, and applying our model comparison procedure to recover the model that generated each dataset (see Table S2).

#### Physiological responses decoding

Statistical significance of decoding accuracies was measured using a one-tailed permutation test. For this purpose, we generated a null distribution based on 100 random permutations of the data, permuting each subject’s behavior-derived prediction errors with respect to that subject’s physiological responses. We then applied the full decoding procedure to each permutated dataset and measured the resulting accuracy.

#### Regression Analyses

We used linear regression to test the relationship between reward-prediction-error decoding from the physiological responses to outcomes during an experimental session and mood change following the sessions. For this purpose, we examined how mood changed 4 hr after each experimental session, when subjects were next asked to report their mood. In addition, to control for diurnal variations in mood [51], we examined how mood changed 24 hr following each session. To account for possible ‘regression to the mean’ effects, we included the current level of mood (i.e., during the experimental session) as a control regressor. To control for multiple comparisons for the two physiological source (heart rate or EEG) and the two timescales of mood change (4 or 24 hr), results were considered statistically significant below a Bonferroni-corrected threshold of p=0.0125. A complementary analysis similarly assessed the relationship between reward-prediction-error decoding and subsequent mood change following any integer number of hours between 1 and 24. Here we corrected p values for multiple comparisons across all possible lags using false-discovery-rate (FDR) adjustment [52].

Logistic regression was used to test the relationship between the subjective value of reward during a session in which an image appeared with reward feedback and later choices involving the image. Here the number of observed rewards for each image served as a control regressor. For both types of regression, statistical significance of regression coefficients was computed at the group level using a two-tailed bias-corrected and accelerated bootstrap test [53] with default MATLAB options.

### Data and Software Availability

All experimental data and analysis scripts are available upon request by contacting the Lead Contact, Eran Eldar (e.eldar@ucl.ac.uk).

## Supplemental Information

Document S1. Figures S1–S4 and Tables S1 and S2

Document S2. Article plus Supplemental Information
