# Supplementary material for: Decodability of Reward Learning Signals Predicts Mood Fluctuations
Source: Curr Biol. 2018 May 7;28(9):1433–1439.e7. doi: 10.1016/j.cub.2018.03.038 (PMC5954908; doi:10.1016/j.cub.2018.03.038)
Supplement: Document S1. Figures S1–S4 and Tables S1 and S2 [file mmc1.pdf]

Current Biology, Volume *28*

Supplemental Information

Decodability of Reward Learning Signals Predicts Mood Fluctuations

Eran Eldar, Charlotte Roth, Peter Dayan, and Raymond J. Dolan

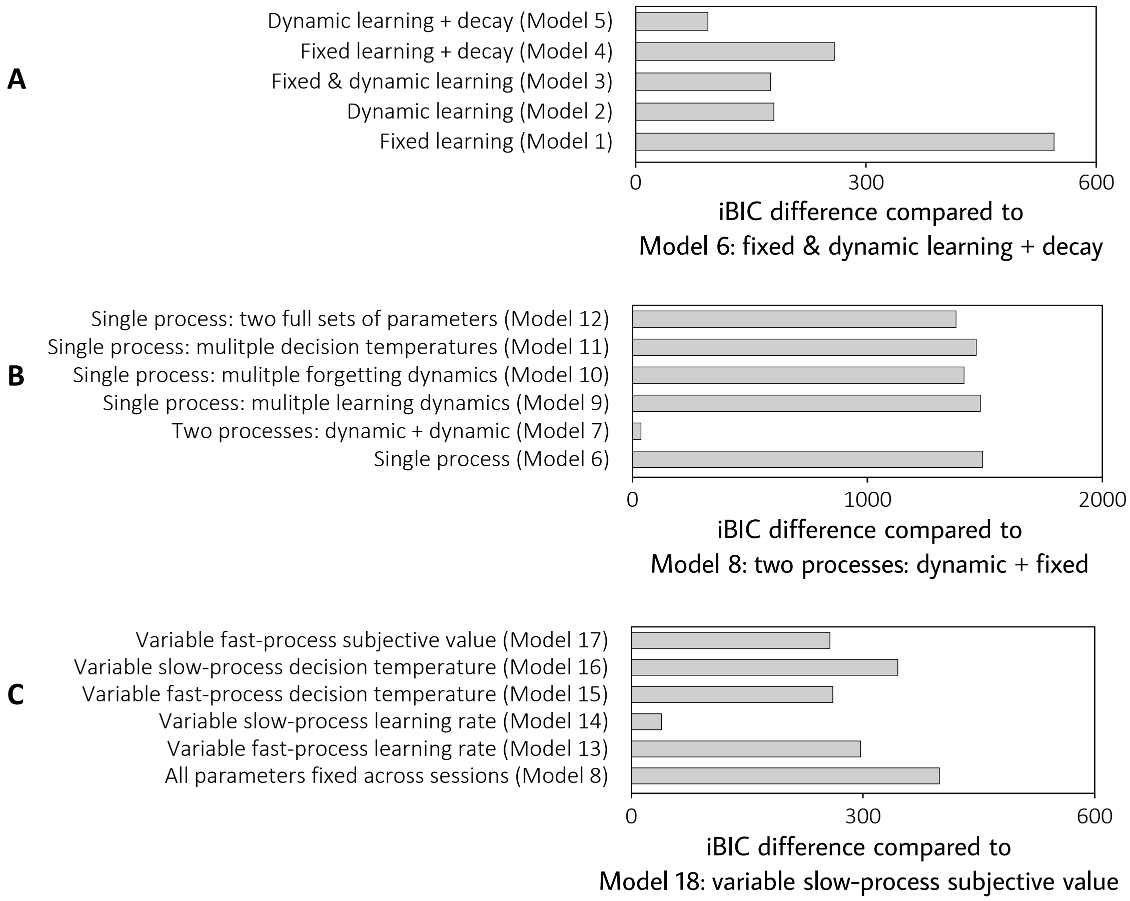

Figure S1. Modeling subjects' choices. Related to Figure 2. n = 10 subjects. To gain insight into subjects' learning processes, we compared multiple models in terms of how well they explained subjects' choices in the task. For each set of models, iBIC scores (integrated Bayesian Information Criterion) are shown in comparison with the best-fitting model. indicates better fit with subjects' choices (see **STAR Methods** for details of all models and model comparison procedure). (**A**) We first tested whether subjects updated their expectations similarly following each outcome ('fixed learning'), gradually reduced their learning rate ('dynamic learning'), or a combination of both ('fixed & dynamic'), as well as whether subjects' expected values decayed as a function of time ('decay'). The best-fitting model ('fixed & dynamic + decay'; iBIC = 22918) included all features. (**B**) We then tested whether subjects' decisions were better explained by assuming that learning involved two sets of expectations, each reflecting outcomes on a different timescale ('Two processes'), whether one of these sets was updated with a fixed learning rate ('dynamic + fixed'), and whether the same dynamics could be captured by models with a single set of expectations but where learning, forgetting and choice selection involve additional parameters that allow for dynamics with multiple timescales (Models 12 to 15). The best-fitting model ('dynamic + fixed'; iBIC = 21428) involved two sets of expectations. (**C**) Finally, we tested whether a subject performed the task similarly in different sessions ('parameters fixed across sessions') or whether subjects' behavior indicated that some aspect of learning or decision making varied from session to session. The best-fitting model ('variable slow-process subjective value'; iBIC = 21070) involved variability in the subjective value of reward. We also tested models with two variable parameters (not shown), but these did not achieve a better score than the best model with a single variable parameter (Model 18).

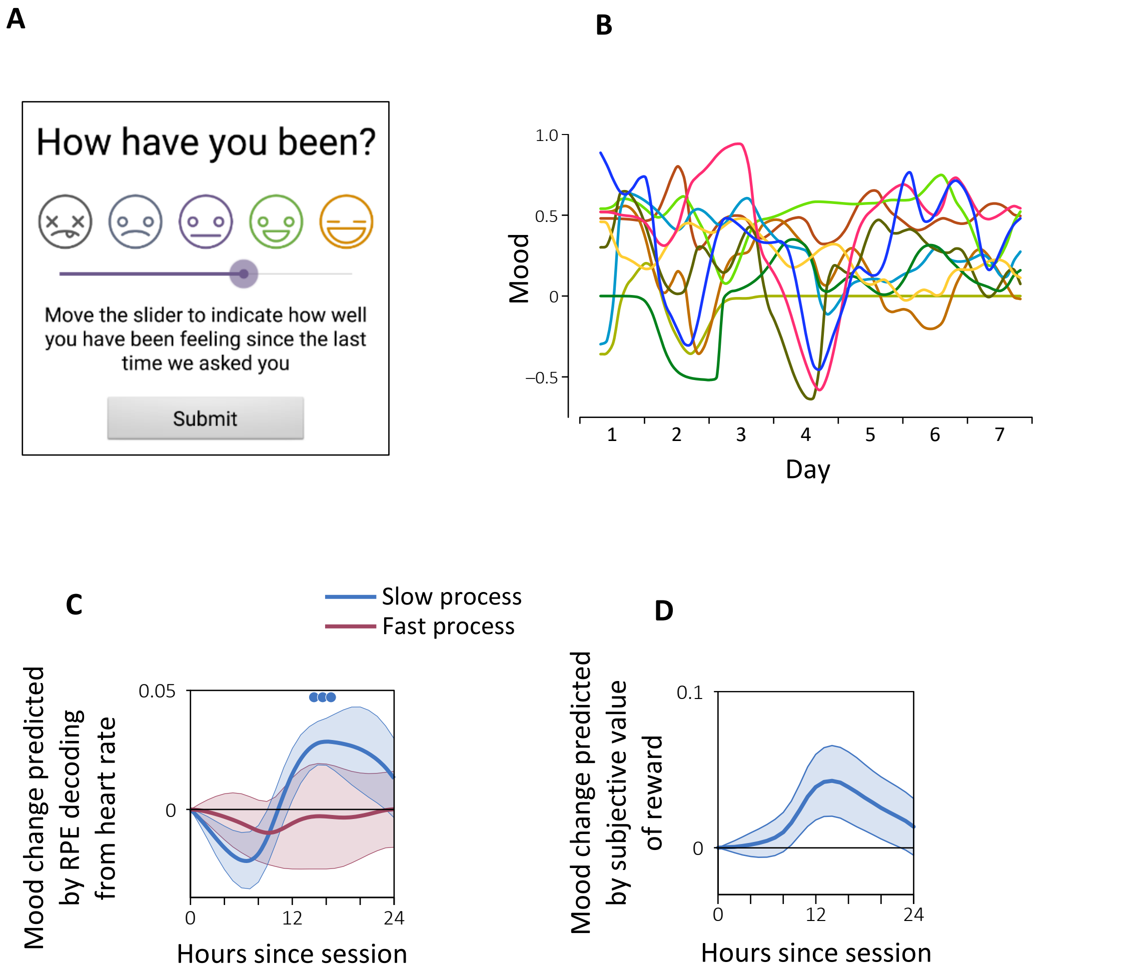

Figure S2. Changes in self-reported mood. Related to Figure 4. (**A**) Mood self-report analog scale. Subjects regularly rated their mood by adjusting the continuous slider. The rightmost end of the slider counted as +1 and the leftmost end as –1. Icons were modeled after the Daylio mobile app [S1]. (**B**) Subjects' self-reported mood over the course of the experiment. 1.0 and –1.0 correspond to best and worst possible mood, respectively. Each line shows one individual subject's mood, integrating all of the subject's self-reports using Gaussian filters (see **STAR Methods**). (**C**) Average change in mood following each experimental session as a function of reward PE decoding from heart rate. (**D**) Average change in mood following each experimental session as a function of reward sensitivity. Sensitivity was inferred from subjects' choices using the computational model via the 'subjective value of reward' parameter ψ. In both panels, magnitude of change is shown per one standard deviation of decoding accuracy / reward sensitivity. ●: difference from zero ($p_{\text{corrected}}$ = 0.04). Shaded areas: SEM.

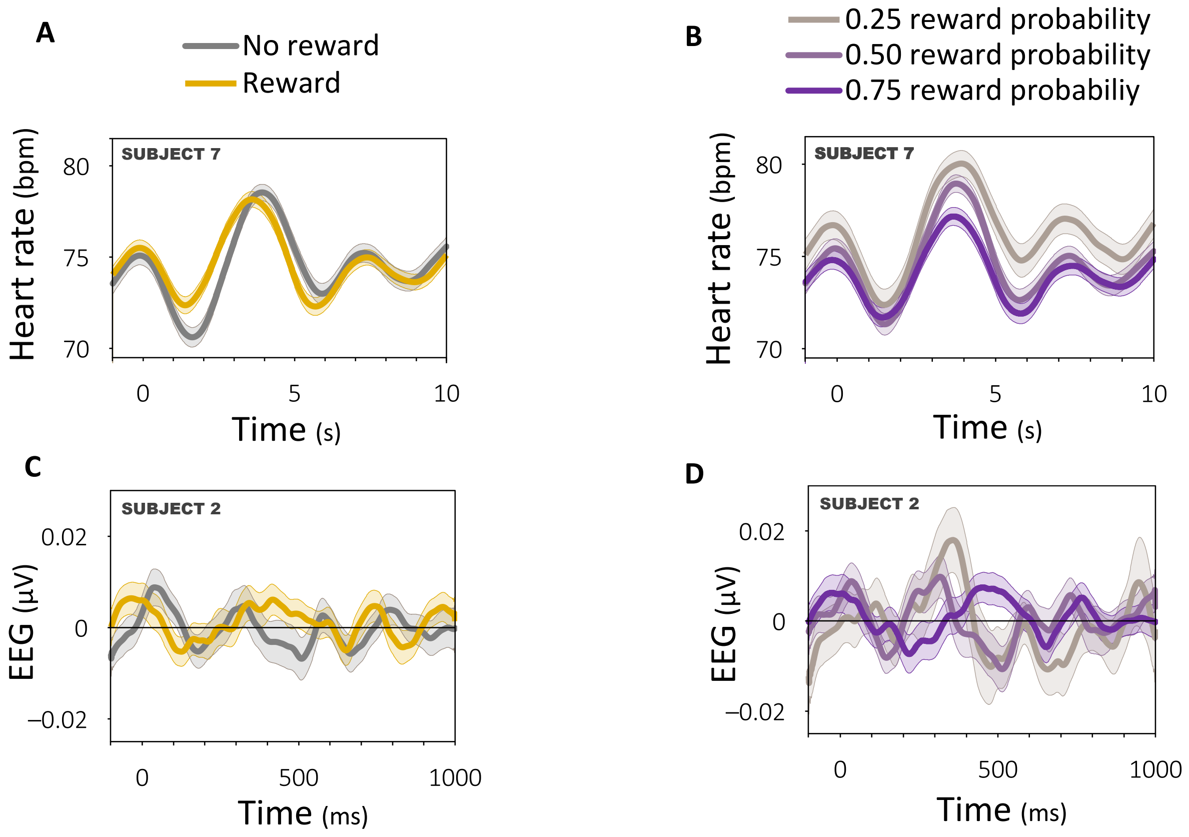

Figure S3. Heart rate and EEG responses to outcomes in the experimental task in two exemplar subjects. Related to Figure 3. Time 0 indicates outcome onset. Shaded areas: SEM (**A**, **C**) Average heart rate (**A**) and EEG (**C**) recorded following reward and no-reward outcomes. (**B**, **D**) Heart rate (**B**) and EEG (**D**) responses to outcomes as a function of the reward probability associated with the chosen image.

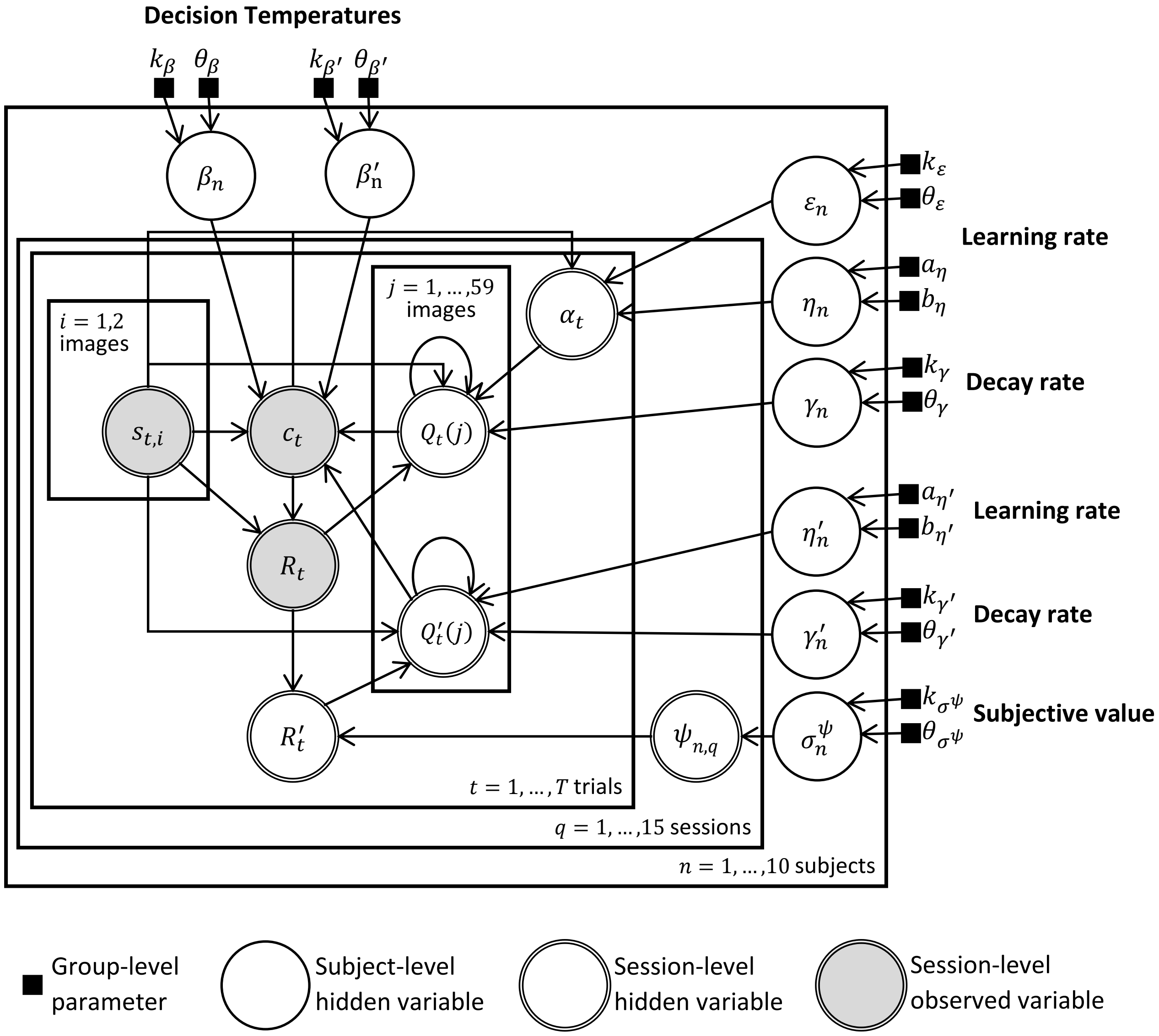

Figure S4. Graphical model of Model 18 (Eqs. 1,2,4,6–8). Related to STAR Methods. Subject n's choice (c) between the images (s) available on trial t of session q, reflect a combination of two sets of expected values (Q and Q'). These two sets of expectations are learned from the same series of choices and outcomes (R) but with different learning and decay dynamics. For the first set (Q), the learning rate (α) decreases as a function of the number of times the chosen image has previously been chosen. For the second set (Q'), the learning rate is fixed (η') and the subjective value of reward (R') varies across sessions. Self-directed arrows indicate that the value of a variable in trial t depends on its value in trial $t - 1$. Session and subject indices are omitted within trials for simplicity.

Model	1	2	3	4	5	6	7	8	9	10	11	12	13	14	15	16	17	18
Fixed learning rate	+			+				+	+			+	+	+	+	+	+	+
Dynamic learning rate		+			+													
Fixed + dynamic learning			+			+	+	+	+	+	+	+	+	+	+	+	+	+
Expectation decay				+	+	+	+	+	+	+	+	+	+	+	+	+	+	+
Two sets of expectations							+	+					+	+	+	+	+	+
Multiple learning rates							+	+	+			+	+	+	+	+	+	+
Multiple decay rates							+	+		+		+	+	+	+	+	+	+
Multiple decision temperatures							+	+			+	+	+	+	+	+	+	+
Variable fast learning rate													+					
Variable slow learning rate														+				
Variable fast dec. temperature															+			
Variable slow dec. temperature																+		
Variable fast subjective value																	+	
Variable slow subjective value																		+

Table S1. Summary of model features. Related to STAR Methods.

Detected Model (columns) / Simulated Model (rows)

	1	2	3	4	5	6
1	5	0	0	0	0	0
2	0	5	0	0	0	0
3	0	0	5	0	0	0
4	0	0	2	3	0	0
5	0	0	0	0	5	0
6	0	0	0	0	0	5,2

Detected Model

	6	7	8	9	10	11	12
6	5	0	0	0	0	0	0
7	0	0	5	0	0	0	0
8	0	0	5,4	0	0	0	0
9	4	0	0	1	0	0	0
10	1	0	0	0	4	0	0
11	0	0	0	0	0	5,2	0
12	0	0	0	0	2,1	0	3,1

Detected Model

	8	13	14	15	16	17	18
8	5	0	0	0	0	0	0
13	1	4	0	0	0	0	0
14	0	0	4,2	0	0	0	1
15	0	0	0	1	0	4	0
16	1	0	0	0	4	0	0
17	0	0	0	1	0	4	0
18	0	0	0	0	0	0	5,2

Table S2. Validation of the model comparison procedure. Related to STAR Methods. We simulated 5 data sets using each model with its parameters fitted to subjects' real choices, and we applied the model comparison procedure to each data set. Each cell shows how many datasets generated by the model indicated on the vertical axis were detected as reflecting the model indicated on the horizontal axis. In red, we indicate cases where a model was detected with a BIC difference (compared to second best model) that was equal or higher than the BIC difference found for subjects' actual data.

Supplemental References

S1. Chaudhry, B.M. (2016). Daylio: mood-quantification for a less stressful you. *mHealth* 2, 34.
